# Systematic Review of Breast Cancer Biology in Developing Countries (Part 1): Africa, the Middle East, Eastern Europe, Mexico, the Caribbean and South America

**DOI:** 10.3390/cancers3022358

**Published:** 2011-05-13

**Authors:** Riyaz Bhikoo, Sanket Srinivasa, Tzu-Chieh Yu, David Moss, Andrew G Hill

**Affiliations:** 1 Department of Surgery, South Auckland Clinical School, University of Auckland, Auckland 1640, New Zealand; E-Mails: sanket.srinivasa@middlemore.co.nz (S.S.); wendells9@gmail.com (T.-C.Y.); andrew.hill@middlemore.co.nz (A.G.H.); 2 Department of Surgery, Middlemore Hospital, Auckland 1640, New Zealand; E-Mail: david.moss@middlemore.co.nz (D.M.)

**Keywords:** breast cancer, breast neoplasm, ethnicity, developing countries, low income countries

## Abstract

There has been no systematic appraisal of ethnicity-based variations in breast cancer (BC) biology amongst women from developing countries. A qualitative systematic review was conducted of breast cancer size, stage, grade, histological type, extra-mammary involvement, hormone receptor status as well as patient demographics. This review includes patients from Africa, the Middle East, Eastern Europe, Mexico, the Caribbean and South America. BC in these regions present at an earlier age with large aggressive tumours. Distant metastases are frequently present at the time of diagnosis. African women have a higher frequency of triple negative tumours. Over half of Middle Eastern women have lymph node involvement at the time of diagnosis. Despite experiencing a lower incidence compared to the Ashkenazi Jewish population, Palestinian women have poorer five-year survival outcomes. The majority of women from Mexico and South America have stage two or three disease whilst over sixty percent of women from Eastern Europe have either stage one or stage two disease. The biological characteristics of BC in the Caribbean cannot be fully assessed due to a paucity of data from the region. BC amongst the developing world is characterised by an early peak age of onset with aggressive biological characteristics. Strategies that improve breast cancer awareness, address amenable risk factors and improve early detection are essential.

## 1. Introduction

Breast Cancer (BC) is the most commonly diagnosed cancer amongst women worldwide [1,2]. There were approximately 1.38 million new cases of BC in the year 2008 and by 2020 this figure is anticipated to escalate to 1.7 million [3,4]. Even though the highest reported prevalence of BC is in developed nations, a significant body of research has found an increasing incidence and poorer survival from BC in developing countries [2-4]. This has been attributed to changes in societal behaviour such as child-bearing practices as well as an adoption of the western lifestyle [5]. Contributing factors also include a lack of awareness of BC especially in the presence of competing infectious diseases with poor access to screening and health care services [6-9]. Many studies have investigated the comparatively poorer survival of indigenous populations within affluent nations and have concluded that differential access to healthcare and socioeconomic status contributes to the inferior outcomes observed [10,11]. However, even after adjusting for these factors, inequalities in mortality still persist and it has been suggested that the discrepancies in survival may be partially explained by ethnicity-related variations in the biological characteristics of BC [12-15].

As a result, there has been growing interest in evaluating the tumour characteristics amongst different ethnic groups. Furthermore, the advancement of genetic testing has placed more importance on the role of genetic factors which may underpin variations in tumour biology. These biological variations may have important consequences for screening, diagnosis and management of BC. However there is a comparative paucity of data from developing countries. Thus, we conducted a systematic review of BC biology in women in developing nations, which we will present as a two part series. In this review we focus on the findings of affected patients residing in Africa, the Middle East, Eastern Europe, Mexico, the Caribbean and South America. In the second part of this review, we will review the characteristics of BC from the Asian Subcontinent and South East Asia [16].

## 2. Methods

Developing countries were defined as per The United Nations Conference on Trade and Development Handbook of Statistics (2008) [17].

### 2.1. Literature Search

The lists of search terms used are listed below:

Breast cancer; Breast malignancy; Breast neoplasm; Breast tumours; Human mammary cancer; Mammary cancer; Mammary carcinoma; Mammary neoplasm; Mammary carcinoma; Ductal carcinoma; Lobular carcinoma; Mastectomy; Infiltrating ductal carcinoma; oestrogen receptors; progesterone receptors; Human epidermal growth factor (HER2); Ethnicity; Race; Nationality.

The databases were searched up to December 2009 using the key terms entered into the following databases: Medline, Ovid, Pubmed, EMBASE, Science Direct, Cochrane database, Web of Science, and EBSCOhost. Manual searching using the Medical Subject Headings (MESH) database with the key term ‘Breast Cancer’ and the country in question (e.g., ‘Nigeria’). Electronic National Cancer Registries were utilised where such information were made available. References of all relevant articles were also screened for further eligible articles. Abstracts published in English were used when full texts were reported in other languages. Where data on BC incidence and/or mortality was not obtainable the International Agency for Research on Cancer (IARC), Cancer Incidence in Five Continents Volumes IX 1998–2000 as well as data from World Health Organisation (WHO) were utilised to gather this information [18].

### 2.2. Selection

Inclusion criteria
  - Studies reporting on the tumour biology of female BC subjects.
Exclusion criteria
  - Studies from developed nations.
  - Papers not listed under the group of developing countries unless either a comparison was made between that country and a developing country, or in cases where data from the developing nations was not readily available then data of expatriates living in developed nations were used.
  - Studies reporting exclusively on male BC.
  - Non-English publications.
Outcome measure
  - Age at diagnosis, tumour size, histological type, grade and stage of cancer and hormone receptor profile.

### 2.3. Validity Assessment and Data Abstraction

The literature search was conducted by two authors (RB and SS) who identified eligible articles and any disagreement was resolved by discussion with the senior authors DM and AGH.

### 2.4. Study Characteristics

Staging of BC was reported using the American Joint Committee of Cancer (6th edition) with histological staging [19]. Alternatively, staging has been reported as local, regional or distant. Tumour grading has been reported using the Scarff-Bloom-Richardson system and is coded from one to three [20].

## 3. Results

**Figure 1.**
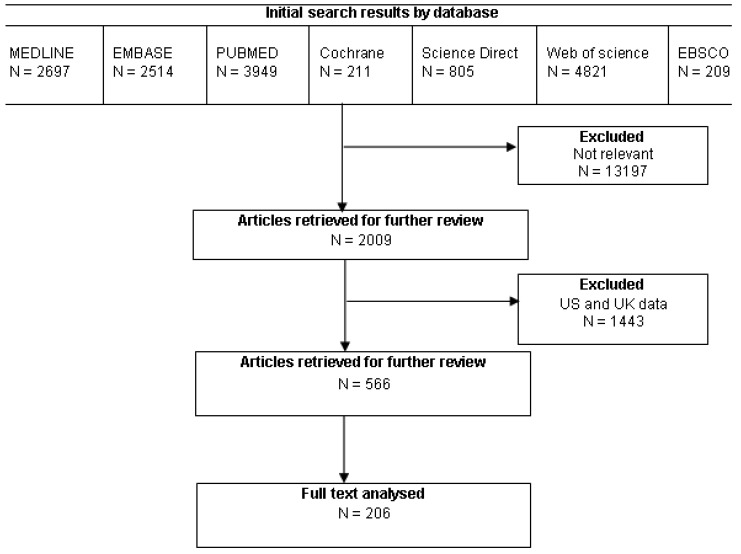
QUOROM flow chart.

The method by which papers were selected for further evaluation has been summarised in Figure 1 [21].

**Figure 2.**
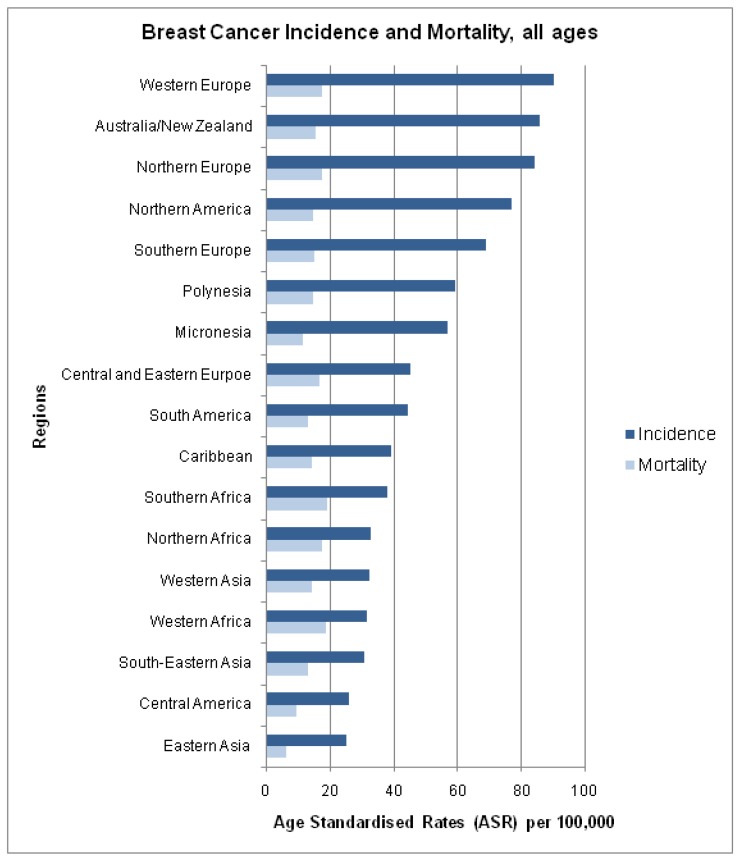
Estimated incidence and mortality from breast cancer in 2008.

The estimated global age standardised incidence and mortality rates of breast cancer in 2008 are presented in Figure 2. Despite having a lower incidence of breast cancer, we find a higher mortality to incidence ratio amongst developing nations (Figure 2).

### 3.1. Africa

An outline of BC biology amongst African nations is presented in Table 1.

#### 3.1.1. Sudan

##### 3.1.1.1. Background and tumour biology

Awadelkarim *et al.* compared the clinico-pathological characteristics of BC between Sudanese and Italian women and found the former presented at a younger age (52 years *vs.* 63 years), had larger tumours (48 mm *vs.* 22 mm), more grade three tumours [68% (78/114) *vs.* 21% (25/120)], more stage three/four disease [38% (33/88) *vs.* 9% (12/137)] and were more likely to have nodal involvement [90% (26/29) *vs.* 36% (40/110)] [22].

##### 3.1.1.2. Hormone receptor status

Sudanese women had less oestrogen receptor (ER+) positive tumours [64% (73/114) *vs.* 83% (114/138)]. However, there were no significant differences in progesterone receptor (PR) positivity [67% (76/114) *vs.* 72% (100/138)], human epidermal growth factor receptor (Her-2/neu) positivity [18% (20/114) *vs.* 10% (14/138)] or combined hormone receptor status (ER and PR considered together) or BC subtypes (*i.e.*, Luminal A and B, basal-like and unclassified) [22].

#### 3.1.2. Nigeria

##### 3.1.2.1. Background

Gukas *et al.* showed women in Jos (Nigeria) presented with BC 21 years earlier than a reference population of women from Norfolk UK (43 years *vs.* 64 years) [29].

##### 3.1.2.2. Tumour biology

Ipkatt *et al.* compared the biological differences of BC between Finnish (n = 285) and Nigerian (n = 300) patients [23]. Nigerian women had less tubular differentiation and had a higher mitotic index to apoptotic index ratio on histology compared to Finnish women [23].

##### 3.1.2.3. Hormone receptor status

In a sample of 129 breast specimens, Ipkatt *et al.* found 24% were ER+ and 14% were PR+ [30]. Gukas *et al.* reviewed 178 specimens and found 25% and 28% were ER+ and PR+ respectively [31]. Using stringent fixation methods, Adebamowo *et al.* found that in contrast to the above studies, most tumours were ER/PR positive, as shown in Table 1 [25]. Amongst women younger than 50 years, 67% were ER+ and 62% PR+ while amongst women older than 50 years, 77% were ER+ and 65% PR+ [25]. 80% were negative for Her-2 while 78% of tumours were luminal type A, three percent were luminal type B, 16% were basal type and four percent were Her-2+/ER− subtype [25]. Hormone receptor status was associated with tumour grade but not with stage at presentation [25]. Huo *et al.* found that most tumours were triple negative for hormone receptors (ER/PR/Her-2) and that these findings reflected inherent biological characteristics rather than poor antigen retrieval related to inadequate tissue fixation [24]. Amongst 378 patients, they showed that 27% of tumours were basal like, 27% were of Luminal A subtype, 15% were Her-2 positive/ER negative and 2% were of the luminal B subtype [24]. Furthermore, Luminal A subtype tumours were less likely to have Ki67 expression (a marker of proliferation) compared to other subtypes [24]. The p53 mutation was more likely to be present among luminal B, Her-2+/ER- and basal-like subtypes [24].

#### 3.1.3. Kenya

##### 3.1.3.1. Background

For the period 2003 to 2006, BC was the leading cancer amongst Nairobi women accounting [32].

##### 3.1.3.2. Tumour biology

Bird *et al.* reported on the results of 129 cases of BC and reported a mean age of 48 years [26]. The histological grade was reported in 114 cases of which 50% were grade three [26]. Cancer stage is shown in Table 1 [26].

Alterman *et al.* reported on the biological characteristics of 118 patients (89% female) between 1993 and 1997 [33]. The mean age was 51 years [33]. The histological grade was reported in 28 premenopausal cases (<50 years) of which 54% were grade three and 39% were grade two [33].

##### 3.1.3.3. Hormone receptor status

Bird *et al.* found 66% (79/120) of cases were ER−/PR−, 24% (29/120) were ER+, 34% were either ER− and/or PR− (41/120) and 10% were ER− but PR+ (12/120) [26]. The authors reported that ER/PR positivity was not associated with stage and not related to age, parity, menopausal status, or node metastases [26].

#### 3.1.4. Tunisia

##### Tumour biology

Maalej *et al.* reported on the biological features of 1437 cases of BC (Table 1) [27]. Nodal involvement was reported in 57% of cases, and of these 20% had greater than 10 nodes involved [27].

#### 3.1.5. Tanzania

##### 3.1.5.1. Tumour biology

In a series of 50 women, Amir *et al.* found 76% had stage IIIb disease whilst none had stage one disease [34]. Metastatic disease, constituted 10% of all cases [34]. All tumours were IDC [34].

##### 3.1.5.2. Hormone receptor status

Mbonde *et al.* assessed tumour markers in 60 patients (Table 1); 33% of cases showed expression for ER, while PR+ tumours were reported in 18%. Rates of expression for Ki-67 (15%), p53 mutation (30%) and bcl-2 (44%) were similar to that reported amongst developed countries [28]. The anti apoptotic protein bcl-2 was strongly co-expressed with ER+ and PR+ tumours [28].

#### 3.1.6. Zimbabwe

##### Background

An analysis of 84 patients treated for breast carcinoma found two age peaks of presentation at between 35 to 40 years and 60 to 65 years [35]. Late presentation was observed in 84% of patients with no further details available.

#### 3.1.7. Democratic Republic of the Congo

##### Background and tumour biology

Kenda *et al.* reported on 134 cases of BC with a mean age of 47 years [36]. IDC accounted for 69% of cases while 96% were found to have either stage three or four disease [36].

#### 3.1.8. South Africa

##### 3.1.8.1. Background

In 2001, BC was the leading female cancer amongst asian and coloured women and the second leading cancer amongst white and black women (13%) [37]. A study of 2130 patients found black women were diagnosed with BC a decade earlier than white women (50 years *vs.* 60 years respectively). The authors attributed this disparity to the difference in the age structure of the two populations (mean age of 26 years *vs.* 35 years respectively) [38].

##### 3.1.8.2. Tumour biology

Stage three and four cancer was reported in 47% and 36% of black patients respectively compared to 25% and 19% of white patients. [38] Black patients had a poorer prognosis within each stage compared to whites. However, using multivariate analysis the authors found that race itself did not independently predict survival [38]. Compiled data from four tertiary hospitals in South Africa for the period 1970 to 1997 reported 8411 new cases of BC of which one third of patients were black [39]. 78% of black women had either stage three or four disease compared to 31% of non-black women [39].

#### 3.1.9. Madagascar

##### Background and tumour biology

In a retrospective analysis of 373 BC cases between 1995 and 2001 found the mean age at diagnosis was 48 years [40]. IDC was the most common histological type with 30% of tumours measuring greater than twenty millimetres (T2). 66% of cases were reported as grade three [40].

### 3.2. Middle East

The tumour characteristics of BC from the Middle East have been outlined in Table 2.

#### 3.2.1. Israel/Palestine

##### 3.2.1.1. Background

The Jewish population in Israel has one of the highest rates of BC in the world [6]. Roa *et al.* reported that genetic mutations in specific BRCA 1 (185delAG and 5382insC) and BRCA2 (6174delT) genes predispose to hereditary BC amongst the Ashkenazi Jewish population [50]. The combined prevalence of these three mutations is 2.5% [51]. Comparatively less is known regarding the BC biology amongst the Palestinian women living in Israel. For the period 1970 to 1995, the reported incidence of BC amongst Palestinian women increased by 94% compared to 32% for Jewish women [52].

##### 3.2.1.2. Tumour biology

Nissan *et al.* found that compared to Jewish women [Ashkenazi (A) and Sephardic women (S)], Palestinian (P) patients presented at an earlier age (P = 52 *vs.* A = 56 *vs.* S = 53), with larger tumours (P = 39 mm *vs.* A = 25 mm *vs.* S = 31 mm) and had significantly worse five year disease free survival figures (P = 50% *vs.* A = 72%) with the results being significant [41].

##### 3.2.1.3. Hormone receptor status

Nissan *et al.* found no difference in expression of ER and PR with 78% of Palestinian women being ER+ and 72% PR positive (Ashkenazi women: 77% ER+ and 71% PR+) [41].

#### 3.2.2. Egypt

##### 3.2.2.1. Background

BC accounted for 18% of all newly diagnosed proven malignancies in women for the period 2003–2004 [53]. The median age of presentation was 49 years [53].

##### 3.2.2.2. Tumour biology

Data from National Cancer pathology registry for 2003–2004 reported that 85% of tumours were IDC while 6% were lobular cancer [53]. The mean tumour size was 32 mm [53]. Grade one tumours constituted 1% of cases while 84% and 15% were grade two and three respectively [53]. Seventy percent of patients had lymph node metastasis at presentation [53].

##### 3.2.2.3. Hormone receptor status

Fifty eight percent of patients were hormone receptor positive with 44% ER+/PR+, 9% ER+/PR− and 5% ER−/PR+ whilst 45% of patients were Her2 (2+) [53].

#### 3.2.3. Saudi Arabia

##### 3.2.3.1. Background

BC was the leading cancer amongst Saudi women in the year 2005, accounting for 24% (932/3834) of all newly diagnosed malignancies with a median age at diagnosis of 46 years [42].

##### 3.2.3.2. Tumour biology

The National Cancer registry reported the distribution of cancer stage as being regional in 45% of cases, localised in 25% of cases, and distant in 12% of cases [42]. Ezzat *et al.*, in an analysis of 801 Saudi women for the period 1986–1991, found 26% (206/801) had stage four cancer [43]. Of the 595 patients with cancer stages one to three (Table 2), 67% were lymph node positive with 34% having greater than four lymph nodes involved [43]. Ibrahim *et al.* identified a median age at presentation of 42 years with 78% younger than 50 years [44]. Nodal involvement was present in 67% of patients [44].

##### 3.2.3.3. Hormone receptor status

Ezzat *et al.* reported that 33% of cases were ER+ and 28% (n = 271) were PR+ [43]. In a further study of 145 patients, positive Her-2 expression (3+) was observed in 28% of patients and correlated inversely with ER status [54].

#### 3.2.4. Iran

##### 3.2.4.1. Background

The National cancer registry in Iran reported 4557 new cases of BC in 2004 [45]. Mousavi combined data from 85 papers and showed BC was most prevalent in women aged 40–49 years with 30% of cases younger than 30 years [45].

##### 3.2.4.2. Tumour biology

Mousavi *et al.* found 72% of tumours were greater than 20 mm in size with lymph node involvement observed in 63% of cases [45].

##### 3.2.4.3. Hormone receptor status

Amongst 220 patients, Saatee *et al.* reported positive Her-2 expression (2+) in 57% of cases with an inverse relationship between ER and Her-2 over expression observed [46]. In a study of 114 patients (97% were women), Fallahazad *et al.* reported that 62% were ER+ and 52% were PR+ [55].

#### 3.2.5. Oman

##### 3.2.5.1. Background

In 2008, BC accounted for 22% of all female cancers [56].

##### 3.2.5.2. Tumour biology

Data from 2008 found 74% of tumours were IDC while lobular carcinoma accounted for 4% of cases [56]. A retrospective review by Al-Moundhri *et al.* of 152 patients diagnosed with invasive BC reported a mean age at presentation of 49 years of which 48% of patients were premenopausal and 20% were 40 years of age or younger [47]. The mean tumour size was 46 mm [47]. Axillary dissection was performed on 120 patients of which 69% had lymph node metastases [47].

##### 3.2.5.3. Hormone receptor status

Using 72 BC tissue specimens, Al-Moundhri *et al.* found over expression of p53, bcl-2 and Her-2 in 42%, 54% and 19% of cases respectively [57]. Over expression of bcl-2 was correlated with low histological grade and positive ER/PR status [57]. The over expression of p53 was significantly correlated with younger age (<40), pre-menopausal status, poor tumour differentiation, a lack of ER/PR expression and an inverse correlation with bcl-2 expression [57].

#### 3.2.6. Bahrain

##### 3.2.6.1. Background

Fakhro *et al.* reported on the clinical presentation of 117 BC patients with a mean age at presentation of 50 years with 56% of patients younger than 50 years [58].

##### 3.2.6.2. Tumour biology

Fakhro *et al.* reported that lump size (via clinical examination, ultrasound and mammography) was between 20–50 mm in 52% (61/117) of cases and greater than 50 mm in 20% (23/117) [58]. Clinical stage two, three and four cancer were reported in 51% (60/117), 21% (25/117) and 11% (13/117) patients respectively [58]. Only 7% (8/117) of patients presented with clinical stage one disease [58]. Axillary lymph node involvement was reported in 50% (59/117) of women with distant metastases reported in 11% [58].

#### 3.2.7. United Arab Emirates (UAE)

##### 3.2.7.1. Background

BC was the most frequently diagnosed cancer among UAE nationals in the year 2002 accounting for 23% of all female cancers [59]. Women aged 40–49 and 50–60 had the highest frequency (30% each) followed by women aged 30–49 (20%) [59].

##### 3.2.7.2. Tumour biology

IDC accounted for 78% of tumours, 15% were epithelial tumours (not otherwise specified) and 5% were ILC [59]. Regional lymph node involvement was present in 48% and distant metastases were observed in 9% [59].

#### 3.2.8. Cyprus

##### Background and tumour biology

Between 1998 and 2001, 44% of Cypriot women were diagnosed with BC before the age of 55 [60]. Data from 1062 cases of BC found 81% had IDC on histology while ILC and Adenocarcinoma accounted for seven percent of cases each [60].

#### 3.2.9. Jordan

##### 3.2.9.1. Background

BC accounted for 31% of all female cancers for the period 1996–2002 [61].

##### 3.2.9.2. Tumour biology

For the period between 1996 and 2001, there were 2930 cases of female BC [60]. Of these 94% had been confirmed histologically of which 82% were IDC followed by lobular carcinoma (7%) and Adenocarcinoma (5%) [60]. Sughayer *et al.* reported on 267 cases of which 90% were IDC while 8% were ILC [62]. Of the tumours reported as IDC, 68% were grade three, 30% were grade two and 3% were grade one [62]. Almasri *et al.* examined 91 cases of BC and found a median age of 48 years with 57% (50/88) occurring in patients younger than 50 years [63]. IDC constituted 84% (76/91) of which 45% (34/76) were grade two and 49% (37/76) grade three [63].

##### 3.2.9.3. Hormone receptor status

Sughayer *et al.* reported on the receptor findings of 240 patients with IDC and found 51% were ER+, 58% were PR+ and 18% were positive for Her-2 (3+) [62]. Furthermore, 44% were ER+/PR+, 7% were ER+PR−, 13% were ER-PR+ and 36% were ER−PR− [62]. Almasri *et al.* found 24% (22/91) of cases had over expression of Her-2 [63]. Of patients less than 50 years, 34% (17/50) were positive for Her-2 (3+), 42% were ER+ (21/50) and 48% (24/50) were PR+ [63]. In those greater than 50 years, 13% (5/38) were Her-2 (3+), 68% (26/38) were ER+ and 58% (22/38) were PR+ [63].

#### 3.2.10. Kuwait

##### Background and tumour biology

Motawy *et al.* described the pathological characteristics of BC from 823 patients between 1993 and 1998 [48]. The median age was 45 years [48]. The mean tumour size was 38 mm with 26% of tumours reported greater than 50 mm [48]. Fifty five percent of cases had lymph node involvement while distant metastases were reported in seven percent of cases [48]. Thirty five percent of tumours were poorly differentiated [48].

#### 3.2.11. Lebanon

##### 3.2.11.1. Background

Data from the National Cancer Registry for the year 2003 found BC accounted for 42% of all female cancers [64]. El Saghir reported on 2,673 cases of female BCs and found a mean age of 50 years with 21% of women diagnosed before the age of 39 years [65].

##### 3.2.11.2. Tumour biology

Data from the National Cancer Registry in 2003 found 83% (1403/1699) were IDC, 6% (109/1699) were lobular carcinoma and 5% were (88/1699) Adenocarcinoma [64]. Chalabi *et al.* reported on 180 French and Mediterranean (Lebanon, Tunisia and Morocco) BC patients and found the latter group tended to be 10 years younger at the time of diagnosis [66]. Furthermore, they were more likely to demonstrate a more aggressive tumour phenotype as evidenced by a greater frequency of grade three tumours and with greater lymph node involvement [66].

##### 3.2.11.3. Hormone receptor status

Abadjian *et al.* found 43% (18/42) of women were positive for ER and PR [67]. 49% (19/39) of tumours were positive for the Ki-67 antigen [67]. Chalabi *et al.* showed Mediterranean BC patients had an up-regulation of cytokeratins KRT8 and KRT1, suggesting a greater frequency of luminal B subtypes compared to tumours observed in France which are frequently Luminal A type [66].

#### 3.2.12. Turkey

##### Background

In 2005, BC accounted for approximately 36% of female cancers in Turkey [68]. Discrepancy exists in the frequency of BC between the eastern (20/100,000) and western regions (50/100,000) with the former having a greater frequency of locally advanced BC (50% *vs.* 20%) [49,69]. As of March 2007 there were 9509 registered cases of BC of which 99% were women [49]. Sixty three percent of women were menopausal while 17% were under the age of 40 years [49]. Data on tumour biology are presented in Table 2.

### 3.3. Eastern Europe

An overview of BC stage amongst developing nations within Eastern Europe is reported in Table 3.

#### 3.3.1. Croatia

##### 3.3.1.1. Background

In 2005, BC accounted for 24% of all female cancers with an ASR of 58 per 100,000 [73].

##### 3.3.1.2. Tumour biology

Bezić *et al.* reported on 2141 cases and found a mean tumour size of 25 mm [74]. The commonest histological types were IDC (70%) followed by ILC (11%) [74]. Forty four percent of tumours were reported as grade two with lymph node involvement in 42% of cases [74]. Data from the Dubrovnik County in 2007 reported that 14% of tumours were carcinoma *in-situ* [75]. 42% of BCs were less than 10 mm in size, 51% were reported as grade one and less than 25% of cases had axillary node involvement [75]. In 2005, 75–80% of cases were clinical cancer stage 0-IIA while 20–25% were stage IIB-IV [75].

##### 3.3.1.3. Hormone receptor status

Bezić *et al.* found 81% of tumours were hormone receptor positive [74]. Vrbanec *et al.* reported an increase in the frequency of ER positive (52% to 62%) tumours and a decrease in PR positive tumours (56% *vs.* 53%) between 1990 to 2002 [76]. Sixty eight percent of ER+ tumours were reported in the 70 to 79 year age group while 56% of PR+ tumours were observed in those aged 40 and 49 years [76].

#### 3.3.2. Bulgaria

##### Background and tumour biology

According to the Bulgarian National Cancer Registry for the year 2004, BC was the leading female malignancy accounting for 24% of newly diagnosed cases [71]. Fifty one percent (1813/3548) of new cases occurred within the 55–74 age group while 22% (792/3548) were reported in women less than 49 years [71]. Seventy percent (2272/3249) of morphologically confirmed cases were IDC while 14% (468/3249) were lobular carcinoma [71].

#### 3.3.3. Ukraine

Data from the Ukraine Cancer Registry in 2007 reported 15,321 new cases and 7556 deaths from BC [72]. Cancer stage has been reported in Table 3 [72].

#### 3.3.4. Armenia

BC is the leading cause of cancer death among Armenian women with 700 new cases diagnosed and 120 deaths each year [77]. No information was available on tumour biology.

#### 3.3.5. Kazakhstan

Igisinov *et al.* reported on 28,707 cases of BC between 1999 and 2008 and found an ASR of 33 per 100,000 [78]. Fifty two percent of patients were between the ages 40–59 years and 18% were greater than 70 years [78]. No information was available on tumour biology.

#### 3.3.6. Kyrgyzstan

Igisinov *et al.* reported on 1,233 patients from Kyrgyzstan between 1995 and 2002 of which 43% (524/1,233) were Kyrgyz, 35% (425/1233) were Russian and 9% (108/1233) were Uzbeks [79]. Russian women had a higher annual standardised incidence rate (27/100,000) compared to Kyrgyz (10/100,000) and Uzbeks (10/100,000) patients. However, Kyrgyz women had an earlier mean age of diagnosis at 39.9 years compared to Uzbeks women at 40.3 years and Russian women at 42.3 years [79]. No information was available on tumour biology.

### 3.4. Mexico

#### 3.4.1. Background

In the year 2006, BC became the leading cause of female cancer deaths in Mexico. [80] Between 2000 and 2006, 50% of cases occurred in women under the age of 50 [81].

Rodríguez-Cuevas *et al.* reported on 96,828 women who had mammograms between March 2005 and December 2006 of which 0.2% had BC [82]. The mean age of affected cases was 54 years with 69% of women less than 60 years of age [82].

#### 3.4.2. Tumour biology

Data from the ministry of health found five to 10% of cases were detected in the early stage (Stage zero to one) [81]. Rodríguez-Cuevas *et al.* found 29% of cases detected through mammography were stage one, 42% were stage two and 27% were stage three [82]. In contrast, the results of 2245 women treated for BC showed 25–40% had stage one or two disease while 57% had either stage three or four cancer [82]. Salazar *et al.* reported on the tumour characteristics of 192 women with invasive BC of which 81% (156/192) of tumours were IDC while 19% (36/192) were ILC [83]. All patients had at least clinical stage two or three cancer [83]. Of the patients with IDC, 84% had stage three disease while 70% of all patients with ILC had stage three cancer [83]. Of the patients that had IDC, axillary node involvement was observed in 24% and 23% of premenopausal and postmenopausal women respectively and in 19% for both pre and postmenopausal women with ILC [83].

#### 3.4.3. Hormone receptor status

Salazar Esquivel *et al.* reviewed the ER and PR receptor status in a selection of pre- and postmenopausal patients with clinical stage three IDC [84]. They found 25% and 26% of pre and post menopausal women were ER+ PR+ while 31% and 27% were ER+/PR−, 21% and 16% were ER−/PR+ and 23% and 31% were ER−/PR− respectively [84].

### 3.5. Caribbean

#### 3.5.1. Barbados

##### Background

Hennis *et al.* reported that mortality outcomes from BC amongst the predominately African-Caribbean population of Barbados were comparable to that of African Americans (33/100,000) despite the former having a lower incidence of the disease (78 *vs.* 144/100,000 respectively) [85].

#### 3.5.2. Cuba

##### Hormone receptor status

Alvarez-Goyanes *et al.* showed that 44% (182/412) of tumours were ER+ [86]. Furthermore, 68% (57/85) of cases were Her-2 negative, 14% (12/85) were slightly positive for the Her-2 receptor (1+/2+) and 19% (16/85) were strongly positive (3+) [86].

#### 3.5.3. Jamaica

##### 3.5.3.1. Background

Data from Kingston and St Andrews for the period 1998–2002 found the incidence of BC was 40 per 100,000 [87].

##### 3.5.3.2. Tumour biology

The Jamaican Breast Disease Study described the clinicopathologic profile of breast disease in a sample of 1189 Jamaican women [88]. Although the majority of patients had benign disease, 23% of biopsy samples showed malignant changes on histology [88]. IDC was identified in 70% of cases [88].

#### 3.5.4. Trinidad and Tobago

##### 3.5.4.1. Background

There were 764 cases of BC for the period 2000–2002, which accounted for 15% of all cancers [89]. Dindyal *et al.* found that African-Caribbean women represented 54% of new cases, followed by Indian-Caribbean women at 35% and mixed races at 11% [90]. Patients aged 53 to 59 years were most affected [90].

##### 3.5.4.2. Tumour biology

Dindyal *et al.* showed IDC accounted for 70% of histological types while ILC accounted for 17% of cases [90].

#### 3.5.5. Panama

##### Background and tumour biology

The median age at presentation in a series of 31 females was 52 years [91]. Forty two percent of cases had stage one disease, 29% with stage two, 16% with stage IIIA, and 13% with stage IIIB [91].

### 3.6. South America

#### 3.6.1. Brazil

##### 3.6.1.1. Background

The Brazilian Ministry of Health estimated 48,930 new diagnoses of BC for the year 2010 with an estimated incidence rate of 49/100 000 women [92]. For the period 2000–2002 Menke *et al.* showed that 76% (104/139) of cases occurred between the ages of 41 and 70 years [93].

##### 3.6.1.2. Tumour biology

Menke *et al.* reported on 1607 cases between 1972 and 2002 and described a significant reduction in the mean tumour size from 35 mm to 28 mm over this period [93]. For the period 2002–2002, 85% (118/139) of tumours were IDC while 10% (14/139) were ILC [93]. For the same time period grade one tumours were reported in 19% (26/139) of cases, grade two in 46% (64/139) and grade three in 18% (25/139) [93]. Distribution of stage one, two and three was reported as 35% (49/139), 46% (64/139) and 19% (26/139) respectively [93]. The percentage of patients presenting with stage one breast cancer doubled between 1972 and 2002 [93]. Forty-one percent (56/138) had axillary node involvement [93].

##### 3.6.1.3. Hormone receptor status

Menke *et al.* reported that 65% of tumours were ER/PR positive [93].

#### 3.6.2. Columbia

##### Background and tumour biology

Two hundred and twenty cases of BC were reported in Pedro Claver Hospital, Bogotá in the year 2004 with a mean age at diagnosis of 59 years [94]. Eighteen percent of cases were diagnosed before the age of 40 [94]. IDC was reported in 84% of cases and 78% of tumours were greater than twenty millimetres [94]. Stage one or two disease was reported in 63% of cases [94].

#### 3.6.3. Peru

##### Tumour biology

Schwartsmann *et al.* reported on 9005 cases between 1985 and 1997 of which 42% were stage two while 33% were stage three [95].

## 4. Conclusion

Breast cancer amongst the developing world is characterised by an early peak age of onset with aggressive biological characteristics. A combined discussion on the topic is included in the second part of this series [16].

## Figures and Tables

**Table 1.** Africa.

**Country**		**Sudan**	**Nigeria**			**Kenya**	**Tunisia**	**Tanzania**
**Author**		**Awadelkarim** [22]	**Ipkatt** [23]	**Huo** [24]	**Adebamowo** [25]	**Bird** [26]	**Maalej** [27]	**Mbonde** [28]
**N =**		114	285	378	192	129	1437	60
**Mean/Median Age (yrs) at presentation**		52	43	45	-	48	51	52
**Mean/Median Tumour Size (mm)**		48	48	55% (21–40 mm)	-	68 (clinical)	33	70%>51
**Histology**	**IDC**	90%	-	87%	82%	90	87%	78%
	**ILC**	5%	4%	4%	2%	-	-	15%
**Grade**	**1**	1%	55%	17%	9%	16%	9%	25%
	**2**	31%		38%	44%	34%	55%	47%
	**3**	68%	45%	44%	16%	50%	35%	28%
**Stage**	**1**	62%	47% * -	-	5%	8% (Stage 0–1)	-	-
	**2**			-	10%	30%	-	7%
	**3**	38%	53% * -	-	38%	46%	-	63%
	**4**			-	48%	17%	-	30%
**LN+**		90%	-	72%	-	72%	57%	-
**ER+**		64%	-	24%	65%	24%	57%	33%
**PR+**		67%	-	20%	55%	-	54%	18%

* Clinical stage; - Information not available.

**Table 2.** Middle East.

**Author**	**N =**	**Age**	**Histology**		**Stage**				**ER+**	**PR+**
		**Mean Median**	**IDC**	**ILC**	**1**	**2**	**3**	**4**		
**Palestine**										
Nissan *et al.* [41]	65	52	-	-	23%	42%	33%	2%	78%	72%
**Saudi Arabia**										
Cancer Registry [42]	930	46	77%	4%	-	-	-	-	-	-
Ezzat *et al.* [43] *	595	-	82%	-	5%	60%	35%	-	33%	28%
Ibrahim *et al.* [44]	292	42	-	-	9%	44%	30%	16%	-	-
**Iran**										
Mousavi *et al.* [45]	85 papers	-	77%	5%	18%	57%	25%	-	-	
Saatee *et al.* [46]	573	49	88%	6%	6%	55%	22%	14%	52%	
**Oman**										
Al-Moundhri *et al.* [47]	152	49	88%	-	10%	47%	42%	-	58%	53%
**Kuwait**										
Motawy *et al.* [48]	823	45	73%	5%	16%	52%	21%	7%	-	-
**Turkey**										
Ozmen *et al.* [49]	9509	-	-	-	23%	52%	17%	4%	66%	44%

* Patients with stage one to three breast cancer only; - Information not available.

**Table 3.** Eastern Europe.

**Country**	**N =**	**Stage**			
		**1**	**2**	**3**	**4**
**Croatia**					
Rudan *et al.* [70]	21491	12%	49%	28%	11%
**Bulgaria**					
National Registry 2004 [71]	3548	23%	48%	20%	7%
**Ukraine**					
National Registry 2007 [72]	15321	74%		16%	8%
